# Molecular and Biochemical Differences in Leaf Explants and the Implication for Regeneration Ability in *Rorippa aquatica* (Brassicaceae)

**DOI:** 10.3390/plants9101372

**Published:** 2020-10-15

**Authors:** Rumi Amano, Risa Momoi, Emi Omata, Taiga Nakahara, Kaori Kaminoyama, Mikiko Kojima, Yumiko Takebayashi, Shuka Ikematsu, Yuki Okegawa, Tomoaki Sakamoto, Hiroyuki Kasahara, Hitoshi Sakakibara, Ken Motohashi, Seisuke Kimura

**Affiliations:** 1 RIKEN BioResource Center, Ibaraki 305-0074, Japan; rumi.amano@riken.jp; 2 Faculty of Life Sciences, Kyoto Sangyo University, Kyoto 603-8555, Japan; g1748146@cc.kyoto-su.ac.jp (R.M.); i1987024@cc.kyoto-su.ac.jp (E.O.); g1747255@cc.kyoto-su.ac.jp (T.N.); k.kami@cc.kyoto-su.ac.jp (K.K.); ikematsu.shuka@cc.kyoto-su.ac.jp (S.I.); okegawa@cc.kyoto-su.ac.jp (Y.O.); k5774@cc.kyoto-su.ac.jp (T.S.); motohas@cc.kyoto-su.ac.jp (K.M.); 3 RIKEN Center for Sustainable Resource Science, Yokohama 230-0045, Japan; mikiko@riken.jp (M.K.); yumiko.takebayashi@riken.jp (Y.T.); kasahara@go.tuat.ac.jp (H.K.); sakaki@agr.nagoya-u.ac.jp (H.S.); 4 Institute of Global Innovation Research, Tokyo University of Agriculture and Technology, Fuchu 183-8506, Japan; 5 Graduate School of Bioagricultural Sciences, Nagoya University, Nagoya 464-8601, Japan; 6 Center of Plant Sciences, Kyoto Sangyo University, Kyoto 603-8555, Japan

**Keywords:** leaf explant, North American Lake Cress, plantlet, phytohormones, vegetative propagation

## Abstract

Plants have a high regeneration capacity and some plant species can regenerate clone plants, called plantlets, from detached vegetative organs. We previously outlined the molecular mechanisms underlying plantlet regeneration from *Rorippa aquatica* (Brassicaceae) leaf explants. However, the fundamental difference between the plant species that can and cannot regenerate plantlets from vegetative organs remains unclear. Here, we hypothesized that the viability of leaf explants is a key factor affecting the regeneration capacity of *R. aquatica*. To test this hypothesis, the viability of *R. aquatica* and *Arabidopsis thaliana* leaf explants were compared, with respect to the maintenance of photosynthetic activity, senescence, and immune response. Time-course analyses of photosynthetic activity revealed that *R. aquatica* leaf explants can survive longer than those of *A. thaliana*. Endogenous abscisic acid (ABA) and jasmonic acid (JA) were found at low levels in leaf explant of *R. aquatica*. Time-course transcriptome analysis of *R. aquatica* and *A. thaliana* leaf explants suggested that senescence was suppressed at the transcriptional level in *R. aquatica*. Application of exogenous ABA reduced the efficiency of plantlet regeneration. Overall, our results propose that in nature, plant species that can regenerate in nature can survive for a long time.

## 1. Introduction

The ability of plants to regenerate into plantlets has been employed in “cutting,” which is an agricultural and horticultural technique for plant propagation and may include root cutting, leaf cutting, and stem cutting [1]. *Rorippa aquatica* (Brassicaceae) propagates asexually by plantlet regeneration from leaf explants in nature [2,3]. *R. aquatica* is an amphibious plant belonging to the Brassicaceae family and it inhabits rivers and lakesides in North America [4,5]. It is often difficult to study plant species which regenerate plantlets in nature because of their properties (e.g., long term plantlet regeneration and poor genetic information). *R. aquatica* requires only two weeks for plantlet regeneration [2] and it belongs to the same family as *A. thaliana* [5]. Some previous studies have revealed the molecular mechanisms underlying leaf development and plantlet regeneration [2,4].

Plantlets of *R. aquatica* emerge from the cut surface of rosette leaf explants [2]. Interestingly, these plantlets regenerate only at the proximal side of the leaf explant. We demonstrated that *R. aquatica* regenerates by autonomous hormone regulation after leaf cutting from time-course developmental and transcriptome analyses [2]. Rosette leaves of *R. aquatica* maintain polar auxin transport from the distal to the proximal side even after cutting, resulting in auxin accumulation at the proximal side of the leaf explant. This accumulated auxin activates the genes involved in morphogenesis at the proximal side, which subsequently triggers root and shoot regeneration (i.e., development of plantlets). Moreover, we showed that gibberellin is necessary for root regeneration, and that cytokinin can promote shoot regeneration [2].

However, these findings still cannot entirely explain the mechanism of plant regeneration, and the fundamental difference between plant species that can and cannot regenerate plantlets from vegetative organs remains to be determined. Here, we hypothesized that the viability of leaf explants is a key factor determining the regeneration capacity in *R. aquatica* and that the leaf explants of *R. aquatica* can survive longer than those of *A. thaliana*. To test this hypothesis, we analyzed and compared the photosynthetic activity, senescence, and immune response of *R. aquatica* and *Arabidopsis thaliana*.

To evaluate the relationship between the ability of plantlet regeneration and viability of leaf explants, time-course of photosynthetic activity, quantification of endogenous phytohormones, and expression profiles of senescence- and immune response-related genes based on transcriptome data were compared between mature rosette leaves of *R. aquatica* and *A. thaliana*. To further assess the effects of photosynthetic activity, leaf senescence, and immune response on the efficiency of plantlet regeneration in *R. aquatica*, leaf explants of *R. aquatica* were cultured on agar media containing 3-(3,4-dichlorophenyl)-1,1-dimethylurea (DCMU), abscisic acid (ABA), or methyl jasmonate (MeJA).

## 2. Results

### 2.1. Leaf Explants of R. aquatica Are Highly Viable

**Figure 1:**
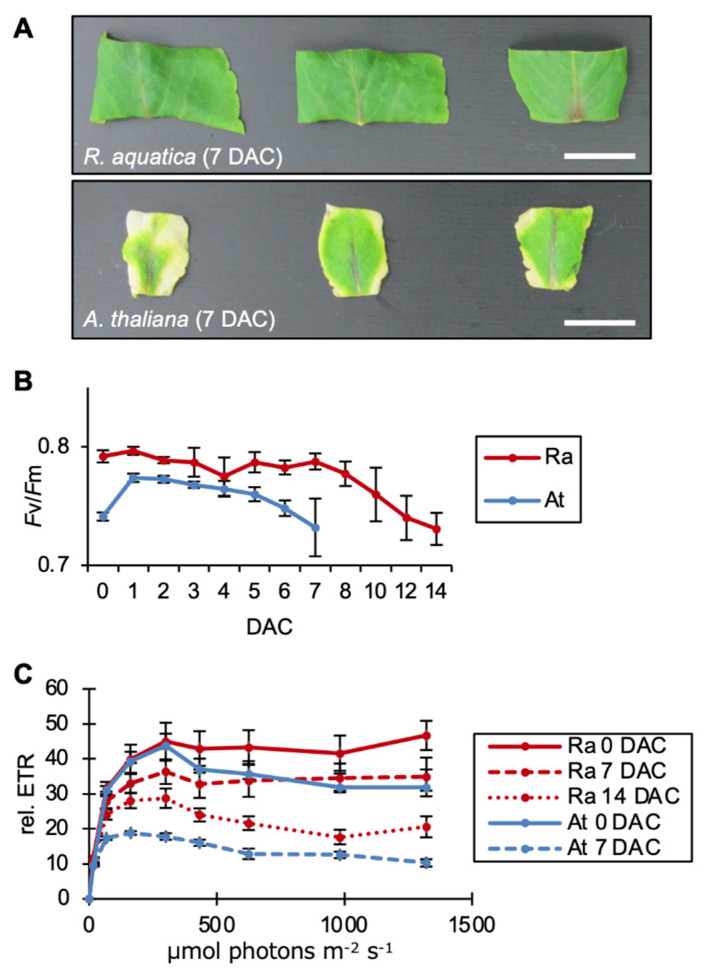
Comparison of the viability of *R. aquatica* (Ra) and *A. thaliana* (At) leaf fragments. (**A**) Leaf explants of *R. aquatica* and *A. thaliana* 7 days after cutting (DAC). Scale bars = 1 cm. (**B**) Time-course measurements of maximum quantum efficiency of photosystem II (*F*v/*F*m). Vertical line and horizonal line indicate *F*v/*F*m and time points, respectively. (**C**) Time-course measurements of light-intensity dependent electron transport rate (ETR). Vertical line and horizonal line indicate relative photosynthetic electron transport rate (rel. ETR) and time points, respectively. As *A. thaliana* explants cultured for more than seven days could not be assessed, measurements were only recorded from 0 to 7 days in *A. thaliana* leaf explants. Dotted and solid lines indicate the expression levels at the distal and proximal sides, respectively. Data are presented as mean ± standard error (SE) (*n* = 5).

To test whether leaf explants of *R. aquatica* survive longer than those of *A. thaliana*, leaf explants 7 days after cutting were compared (Figure 1A). Leaf explants of *R. aquatica* remained green (Figure 1A), whereas those of *A. thaliana* exhibited etiolation at the margin and cut surface, indicating senescence (Figure 1A). These results suggest that *R. aquatica* leaf explants are more viable than those of *A. thaliana*.

To further assess the physiological ability of leaf explants to survive, their photosynthetic activity was measured from 0 to 14 days after leaf cutting. *A. thaliana* leaf explants cultured for more than seven days could not be used for photosynthetic analysis due to plant death; hence, the photosynthetic activity of these explants was measured from 0 to 7 days after cutting. The maximum quantum yield of photosystem II (*F*v/*F*m) of *R. aquatica* remained at approximately 0.8 up to day 7, after which it exhibited a gradual decrease (Figure 1B). In *A. thaliana*, the *F*v/*F*m value began to decrease gradually from day 2 (Figure 1B). The *F*v/*F*m value of *A. thaliana* leaf explants on day 7 (0.732) was nearly equal to that of *R. aquatica* on day 14 (0.731) (Figure 1B). The electron transport rate (ETR) of *R. aquatica* leaf explants exhibited a gradual decrease from day 0 to 7 and then to day 14 (Figure 1C). The ETR of *A. thaliana* on day 7 was lower than that on day 0 (Figure 1C). The ETR of *A. thaliana* on day 7 was lower than that of *R. aquatica* on day 14 (Figure 1C). These results indicate that *R. aquatica* leaf explants maintained their photosynthetic ability 14 days after cutting, when plantlets start to emerge.

### 2.2. Photosynthesis Is Required for the Survival of Leaf Explants and Plantlet Regeneration in R. aquatica

**Figure 2:**
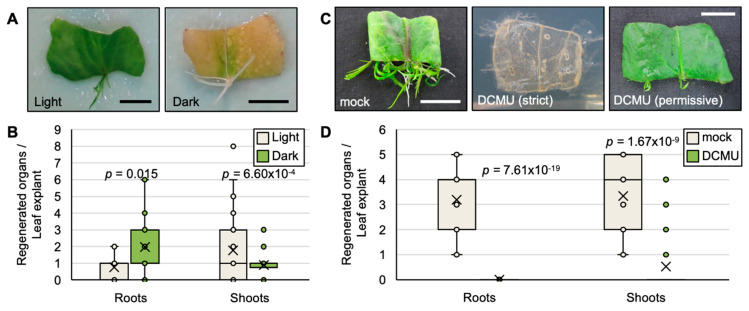
Effects of photosynthetic inhibition on vegetative propagation in *R. aquatica*. (**A**,**B**) Plantlet regeneration under dark conditions. (**A**) Effects on leaf fragments at day 22. (**B**) Quantification of regenerated roots and shoots at day 22 (*n* = 30). Vertical line and horizonal line indicate the number of regenerated organs per leaf explant, and roots or shoots, respectively. (**C**,**D**) Effect of the photosynthesis inhibitor 3-(3,4-dichlorophenyl)-1,1-dimethylurea (DCMU). Mock is dimethyl sulfoxide (DMSO). (**C**) Effects on leaf fragments at day 22. Both strict and permissive effects were observed. (**D**) Quantification of regenerated roots and shoots at day 22 (*n* = 32). Vertical line and horizonal line indicate the number of regenerated organs per leaf explant, and roots or shoots, respectively. Scale bars = 1 cm. The average value is indicated by a cross mark in each box plot. Significant differences were determined by Student’s *t*-test and *p* values indicate exact values.

Photosynthetic activity measurements showed that the leaf fragments of *R. aquatica* could maintain their photosynthetic ability for a longer time. To assess if the photosynthetic activity of leaf explants supports the ability of plantlet regeneration, *R. aquatica* leaf explants were cultured in dark conditions. After 23 days, almost all leaf explants were etiolated (Figure 2A). However, few plantlets were regenerated and also etiolated (Figure 2A). Under dark conditions, shoot regeneration was decreased while root regeneration was increased (Figure 2B).

Furthermore, leaf explants of *R. aquatica* were cultured on agar medium containing DCMU, a photosynthesis inhibitor that blocks electron transfer between the primary (Q_A_) and secondary (Q_B_) quinone electron acceptors on the reducing side of PSII [6]. DCMU exerted two types of effects on the leaf explants, i.e., strict and permissive effects. Almost all leaf explants exhibited discoloration followed by death (strict effect) (Figure 2C); 75% of leaf fragments showed a strict effect. Few leaf explants remained green and regenerated shoots (permissive effect) (Figure 2C,D). These results indicate that maintenance of photosynthesis might be important for plantlet regeneration.

### 2.3. Phytohormones Are Regulated Differently in R. aquatica and A. thaliana Leaf Explants

To assess if the phytohormone levels trigger senescence in leaf explants, endogenous ABA and JA levels in *R. aquatica* and *A. thaliana* leaf explants were quantified over time separately for the distal and proximal sides.

**Figure 3:**
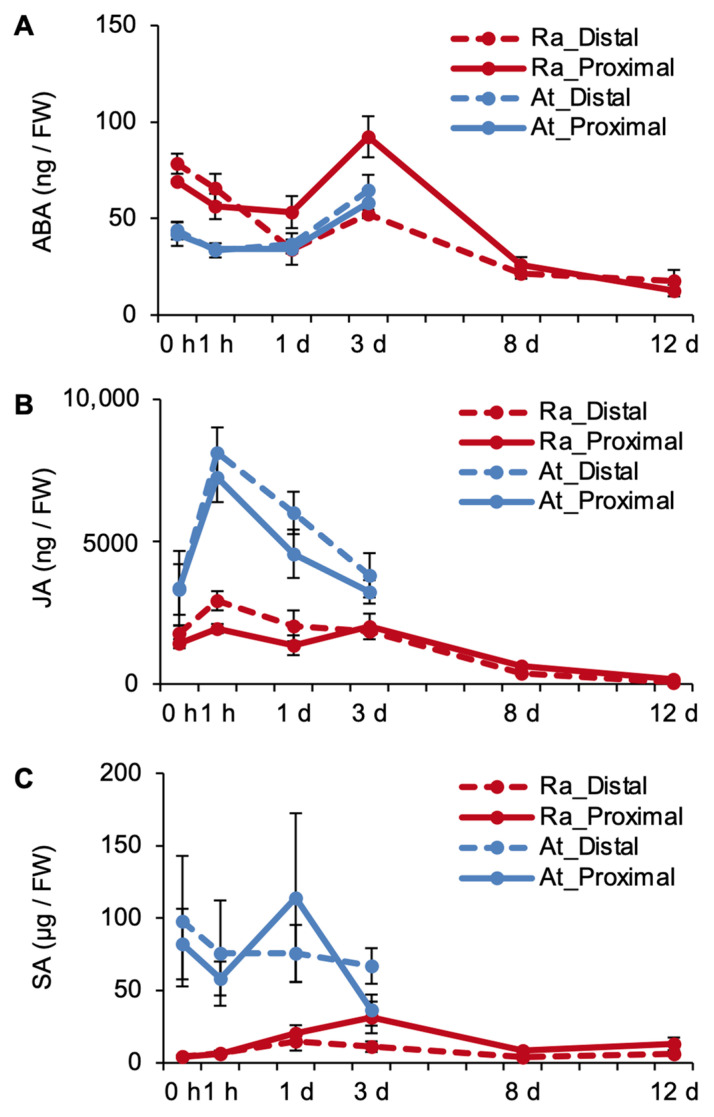
Time-course quantification of endogenous abscisic acid (ABA), jasmonic acid (JA), and salicylic acid (SA) in leaf fragments of *R. aquatica* and *A. thaliana*. (**A**) Quantification of endogenous ABA. (**B**) Quantification of endogenous JA. (**C**) Quantification of endogenous SA. Leaf fragments of *A. thaliana* could only be analyzed only from day 0 to 3. Dotted and solid lines indicate the expression levels at the distal and proximal sides, respectively. The vertical line and horizonal line indicate the amount of phytohormone (FW; fresh weight), and time points, respectively. Data are presented as mean ± SE (*n* = 5).

In *R. aquatica* leaf explants, the ABA level increased at both distal and proximal sides for three days, following which it decreased (Figure 3A). The ABA levels in *A. thaliana* leaf explants also increased up to day 3, at both the distal and the proximal sides (Figure 3A). However, the levels at later time points could not be analyzed due to the death of these explants. JA levels in *R. aquatica* decreased gradually, at both distal and proximal sides (Figure 3B). In *A. thaliana*, JA levels increased rapidly after cutting (i.e., 1 h), and then decreased on day 1 (Figure 3B). Overall, both ABA and JA levels decreased in *R. aquatica*, at both distal and proximal sides of the explants (Figure 3A,B).

Additionally, to test the differences in the immune response of the two explants at the phytohormone level, endogenous SA levels were also quantified in *R. aquatica* and *A. thaliana* leaf explants. The SA level in *R. aquatica* was considerably lower than that in *A. thaliana*, and it did not change over time (Figure 3C).

### 2.4. Senescence and Immune Responses Are Regulated at the Transcriptional Level in R. aquatica Leaf Explants

Phytohormone quantification revealed changes in ABA and JA levels during plantlet regeneration in *R. aquatica* (Figure 3A,B). To analyze how these hormones were regulated at the transcriptional level, RNA-seq transcriptome data of *R. aquatica* and *A. thaliana* leaf explants were used.

**Figure 4:**
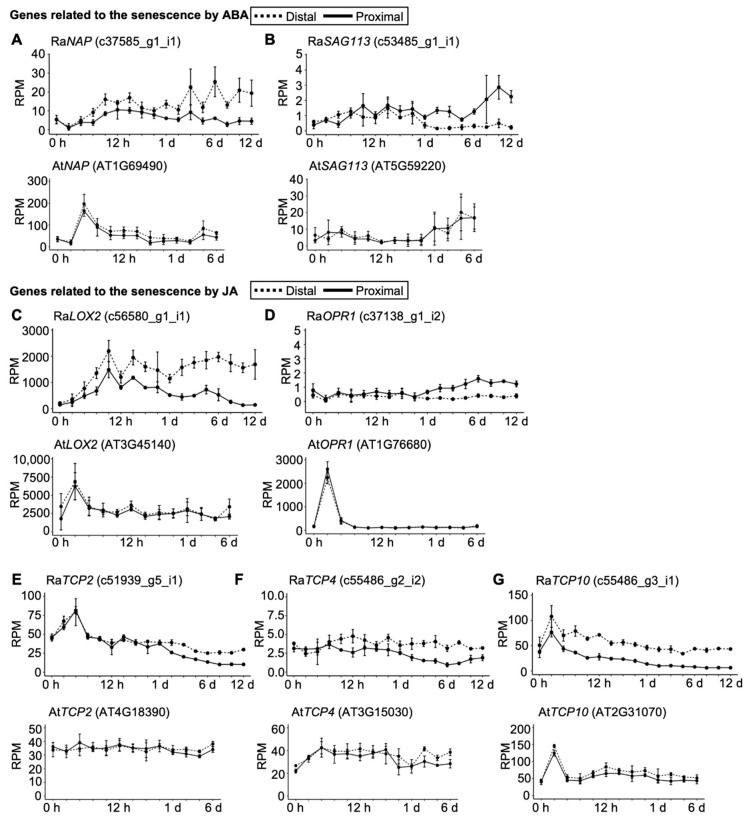
Expression profiles of senescence-related genes and orthologs. (**A**,**B**) Expression profiles of senescence-related genes and orthologs affected by abscisic acid (ABA). (**C**–**G**) Expression profiles of senescence-related genes and orthologs affected by jasmonic acid (JA). Upper and lower panels depict expression profiles of *R. aquatica* and *A. thaliana*, respectively. Dotted and solid lines indicate expression levels at the distal and proximal sides, respectively. Vertical line and horizonal lines indicate reads per million (RPM) and time points, respectively. Data are presented as mean ± SE (*n* = 3).

*NAC-like*, activated by *apatala3/pistillata* (Ra*NAP*), an ortholog of an important positive regulator of leaf senescence in *A. thaliana* and *Oryza sativa* [7,8,9,10], was upregulated only at the distal side of the explant at later time points (Figure 4B). At*NAP* was rapidly upregulated at both the distal and proximal sides after leaf cutting in *A. thaliana* (Figure 4B), indicating earlier activation of the ABA response in *A. thaliana*. *SENESCENCE ASSOCIATED GENE 113* (Ra*SAG113*), on ortholog of a negative regulator of ABA signaling in *A.thaliana* [11], was not changed until day 6 in *R. aquatica*, whereas it was upregulated on day 1 in *A. thaliana* leaf explants (Figure 4A). Additionally, upregulation of Ra*SAG113* was observed only at the proximal side of the explant (Figure 4A).

Although orthologs of JA biosynthesis genes *LIPOXYGENASE* (*LOX*) and *OXOPHYTOSIENOATE-REDUCTASE* (*OPR*) were upregulated at both the distal and the proximal sides at earlier time points, their expression levels remained low at later time points in *R. aquatica* (Figure 4C). *LOX2* was upregulated in *A. thaliana* after 1 h (Figure 4C). Orthologous genes of *TEOSINTE BRANCHED*/*CYCLOIDEA*/*PCF* (*TCP2*), *TCP4*, and *TCP10*, transcription factors related to biosynthesis of JA [12], were downregulated in *R. aquatica* (Figure 4E–G). These results further support the persistence of *R. aquatica* leaf explants from a transcriptional aspect. *OPR1* was upregulated after 1 h in *A. thaliana*, whereas no such change could be detected in *R. aquatica* (Figure 4D).

**Figure 5:**
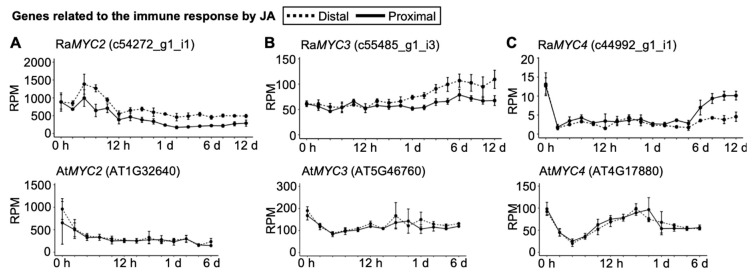
Expression profiles of immune response-related genes and orthologs affected by jasmonic acid (JA). Upper and lower panels depict expression profiles of *R. aquatica* and *A. thaliana*, respectively. Dotted and solid lines indicate expression levels at the distal and proximal sides, respectively. Vertical line and horizonal lines indicate reads per million (RPM) and time points, respectively. Data are presented as mean ± SE (*n* = 3).

An ortholog of a gene which activates JA signaling during saprobe infection in *A. thaliana* [13], Ra*MYC2*, was downregulated following an initial upregulation, whereas it exhibited rapid downregulation in *A. thaliana* (Figure 5A). It was noteworthy that both Ra*MYC3* and Ra*MYC4* were upregulated, whereas they were downregulated in *A. thaliana* (Figure 5B,C).

### 2.5. Exogenous ABA and JA Affect the Efficiency of Plantlet Regeneration

**Figure 6:**
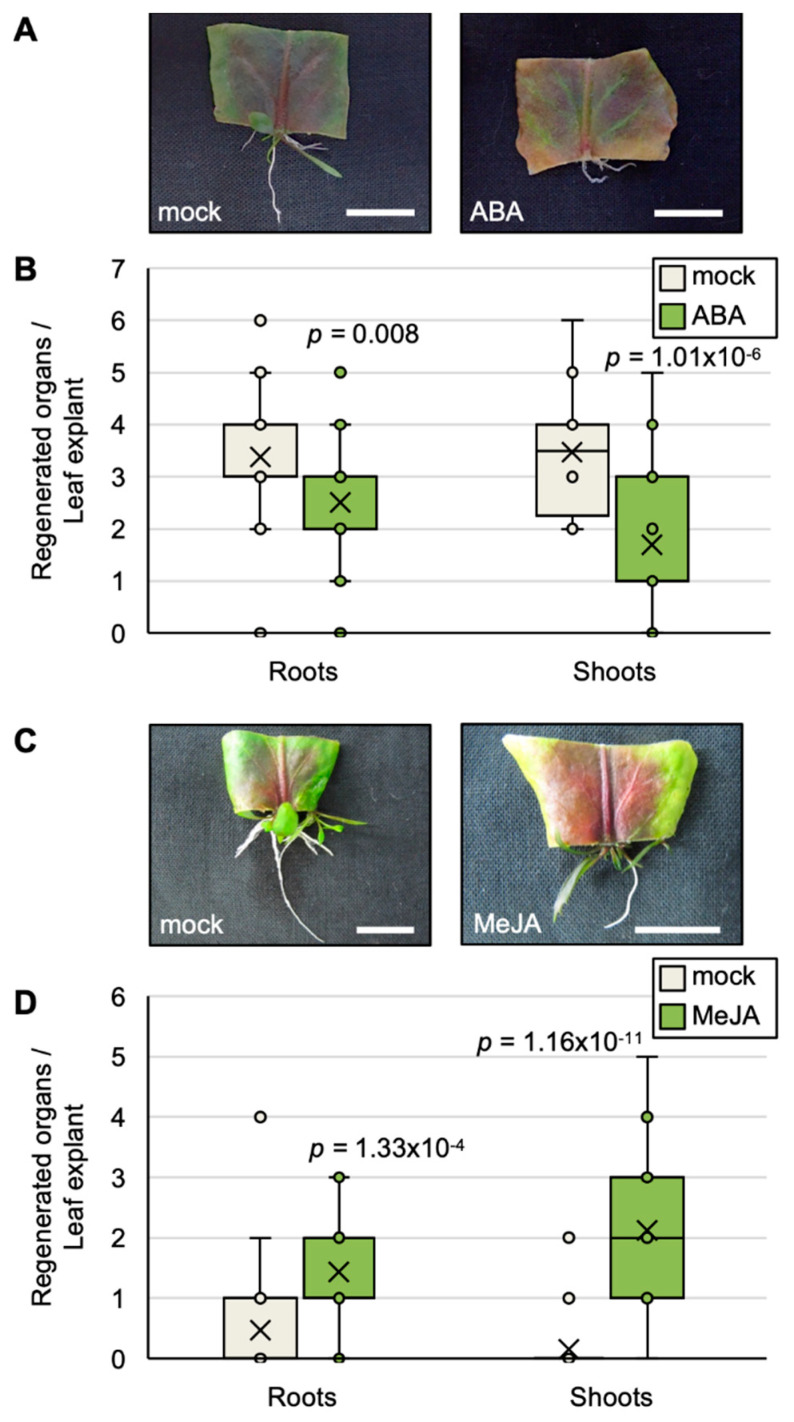
Effects of the exogenous application of abscisic acid (ABA) and jasmonic acid (JA) on vegetative propagation in *R. aquatica*. (**A**,**B**) Effect of ABA on vegetative propagation in *R. aquatica*. (**A**) Leaf fragments cultured on agar medium containing 50 µM ABA at day 22. (**B**) Quantification of regenerated roots and shoots at day 22 (*n* = 32) (**B**). The vertical line and horizonal line indicate the number of regenerated organs per leaf explant, and roots or shoots, respectively. (**C**,**D**) Effect of JA on vegetative propagation in *R. aquatica*. (**C**) Leaf fragments cultured on agar medium containing 5 µM methyl jasmonate (MeJA) at day 22. (**D**) Quantification of regenerated roots and shoots at day 22 (*n* = 32) (**D**). The vertical line and horizonal line indicate the number of regenerated organs per leaf explant, and roots or shoots, respectively. Scale bars = 1 cm. Mock is dimethyl sulfoxide (DMSO). The average value is indicated by a cross mark in each box plot. Significant differences were determined by Student’s *t*-test and *p* values indicate exact values.

To examine effect of ABA and JA on the efficiency of plantlet regeneration, *R. aquatica* leaf explants were cultured on agar media containing either ABA or MeJA. On the ABA-containing medium, the color of the leaf explants changed to red and yellow (Figure 6A), and the number of regenerated organs decreased significantly (Figure 6B). The leaf explants also changed color to red on the MeJA-containing medium (Figure 6C). However, the number of regenerated organs were increased (Figure 6D).

### 2.6. Internal Leaf Structure May Affect the Viability of Leaf Explants

**Figure 7:**
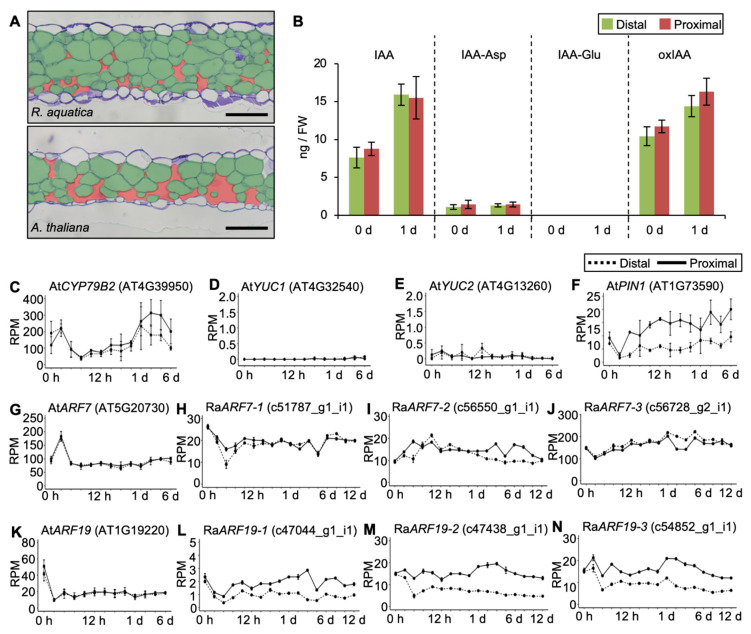
The relationship between internal leaf structure and auxin accumulation during plantlet regeneration (**A**) Internal structure of mature *R. aquatica* and *A. thaliana* rosette leaves. Transverse sections of leaves were stained with 0.1% toluidine blue (*w*/*v*). Mesophyll cells and intercellular spaces were stained green and red, respectively. Scale bars = 200 µm. (**B**) Quantification of endogenous indole-3-acetic acid (IAA) and its metabolites [IAA–Asp, IAA–Glu, and 2-oxindole-3-acetic acid (oxIAA)] at the distal and proximal sides of *A. thaliana* leaf explants 0 and 1 day after leaf cutting. The vertical line and horizonal line indicate the amount of IAA or its metabolites (ng/fresh weight), and day 0 (0 d) or day 1 (1 d), respectively. IAA–Glu was beyond the detection range. No significant differences could be detected between the distal and proximal sides at any time point (Student’s *t*-test). Data are presented as mean ± SE (*n* = 4). (**C**–**N**) Expression profiles of genes related to auxin biosynthesis in *A. thaliana* (**C**–**E**), auxin transport in *A. thaliana* (**F**), and auxin responses in *A. thaliana* and in *R. aquatica;* (**G**–**N**) plotted by transcriptome data. Dotted and solid lines indicate expression levels at the distal and proximal sides, respectively. The vertical line and horizonal line indicate reads per million (RPM) and time points, respectively. Data are presented as mean ± SE (*n* = 3).

Next, to investigate if there are any other factors affecting the viability of leaf explants of *R. aquatica*, the intercellular structure of rosette leaves of *R. aquatica* and *A. thaliana* were compared. The intercellular space was narrower in *R. aquatica* than in *A. thaliana* leaves (Figure 7A).

Furthermore, to explore the differences between plant species that can and cannot regenerate plantlets from leaf explants in nature, the amounts of endogenous auxins and expression profiles of auxin-related genes were analyzed in *A. thaliana*. Similar amounts of indole-3-acetic acid (IAA) and its metabolites were observed at both distal and proximal sides of *A. thaliana* leaf explants (Figure 7B). At*CYP79B2*, one of the auxin biosynthesis genes, was upregulated after day 1 (Figure 7C). Remarkably, *YUCCA* (*YUC*), an auxin biosynthesis gene, was almost unchanged (Figure 7D,E). These suggest that increased endogenous IAA was synthesized via the *CYP79B2* pathway.

The expression profile of the *PIN-FORMED1* (At*PIN1*) gene, encoding an auxin transport protein, was upregulated at the distal side (Figure 7F). *AUXIN RESPONSIVE FACTOR7* (*ARF7*) and *ARF19*, which are auxin responsive genes, were also downregulated in *A. thaliana* (Figure 7G,K), however, expression of Ra*ARF7* and Ra*ARF19* were not downregulated like *A. thaliana* (Figure 7H–J,L–N).

## 3. Discussion

We studied the effect of the viability of detached leaves on the efficiency of plantlet regeneration in terms of photosynthetic activity, leaf senescence, and immune response in *R. aquatica* and *A. thaliana*.

The measurements of photosynthetic activity and the inhibition thereof suggested that the ability of plantlets to regenerate from leaf explants depends on the photosynthetic activity of the explant (Figure 1B,C and Figure 2B,D).

With regard to leaf senescence, although ABA was increased at the proximal side on day 3, it exhibited a decrease from day 8 in *R. aquatica* (Figure 3A). This suggests a mechanism to reduce endogenous ABA levels, even if they increase initially, suppressing senescence in *R. aquatica* leaf explants. JA levels remained low in *R. aquatica* (Figure 3B). It is possible that the leaf explants of *R. aquatica* suppress senescence by maintaining low levels of these phytohormones throughout the explant.

On a molecular level, ABA signaling is upregulated during senescence in many plant species [14]. Some studies have reported that *NAP* is an important positive regulator of leaf senescence in *A. thaliana* and *O. sativa* [7,8,9,10]. Overexpression of At*NAP* and Os*NAP* promotes leaf senescence, and knockdown mutants of these genes exhibit delayed senescence [8,9]. Furthermore, *SAG113*, a negative regulator of ABA signaling, controls water loss in aging *A. thaliana* leaves [11]. *SAG113* is induced by ABA, and the loss-of-function mutant of this gene exhibits delayed leaf senescence [11]. At*SAG113* gene is a direct target gene of At*NAP* transcription factor [15]. Orthologs of these genes in *R. aquatica* were upregulated at time points later than *A. thaliana* (Figure 4A).

JA is another factor promoting senescence [16]. Exogenous JA promotes leaf senescence in wild-type *A. thaliana* [17]. In senescent *A. thaliana* leaves, the transcriptional expression of JA biosynthesis genes, including *LOX* and *OPR*, increases gradually [17]. JA biosynthesis genes are regulated by miR319 (*JAGGED AND WAVY* (*JAW*)) and *TCP* transcription factors [12]. miR319 can repress the expression of *LOX2* and reduce JA levels through degradation of TCP [12]. This results in delayed leaf senescence, which can be rescued by exogenous JA application [12]. Ra*TCP* genes were downregulated during plantlet regeneration (Figure 4E–G). Considering that the expression profiles of these orthologous genes are related to ABA and JA, it is possible that senescence is delayed at the transcriptional level in *R. aquatica* leaf explants.

JA is also known to be involved in plant immune responses [18], and the biosynthesis of JA and SA has previously been reported to be upregulated in *A. thaliana* leaves infected with saprobes and parasites, respectively [19]. The SA level in *R. aquatica* did not change over time (Figure 3C). This suggests that SA is not important for plantlet regeneration. On a molecular level, JA is biosynthesized and the JA receptor CORONATINE INSENSITIVE 1 (COI1) transmits downstream signals via binding to JASMONATE ZIM DOMAIN (JAZ) in leaves infected with saprobes [20,21]. In normal leaves, JAZ binds to JA signaling activators, i.e., the helix–loop–helix (bHLH) transcription factors MYC2, MYC3, and MYC4, and suppresses JA signaling [13]. In conditions inducing JA production, such as saprobe infection, COI1 degrades JAZ via the proteasome system [13], resulting in the binding of MYC transcription factors to the recognition sequence (G-box; CACGTG) and inducing the expression of JA responsive genes [13]. Ra*MYC3* and Ra*MYC4* were upregulated (Figure 5B,C). These results suggest that the immune response is induced by JA, rather than SA, in *R. aquatica* leaf explants.

Additionally, exogenous ABA changed the color of leaf explants and reduced the efficiency of plantlet regeneration (Figure 6A,B). This might result from the promotion of senescence of the leaf explant. This proposes that the suppression of ABA-dependent senescence in leaf explants is important for plantlet regeneration. Reddening of leaf explants on the application of exogenous JA (Figure 6C) might indicate stress from an upregulated immune response rather than from senescence because exogenous JA did not reduce the efficiency of plantlet regeneration. This result proposes that JA has little involvement in senescence. Overall, these results propose the possibility that senescence in *R. aquatica* leaf explants is delayed by the suppression of ABA levels and a delayed ABA response.

In a previous study, we sectioned *R. aquatica* leaf explants [2] and observed narrow intercellular spaces and tightly packed cells, in both palisade and spongy tissues. Therefore, in the present study, we hypothesized that the internal structure of *R. aquatica* leaves is different from that of *A. thaliana*, and that the packed cells allow leaf explants to survive longer by retaining water, a high photosynthetic efficiency, and efficient auxin transport across the membrane. As expected, the intercellular space of *R. aquatica* was narrow (Figure 7A). Additionally, we have previously reported a greater accumulation of auxin and its metabolites at the proximal side of *R. aquatica* leaf explants, when compared with the distal side, on day 1 [2]. To confirm if *A. thaliana* leaf explants accumulate auxins similarly, endogenous auxin levels at the proximal and distal sides of *A. thaliana* leaf explants on day 0 and day 1 after leaf cutting were compared. In *A. thaliana*, IAA and its metabolites were increased at both the distal and proximal sides on day 1 (Figure 7B).

To examine where increased endogenous IAA after day 1 was synthesized, expression profiles of genes related to IAA biosynthesis were analyzed using transcriptome data from *A. thaliana*. At*YUC* was not upregulated in this study using leaf explants from aged leaves of *A. thaliana* (Figure 7D,E). A previous study has reported that *YUC* genes are required for de novo root organogenesis from young leaves of *A. thaliana* [22]. This suggests that upregulation of *YUC* genes depends on aging on an individual level in *A. thaliana*. In addition, our previous study has reported that Ra*YUC2* were upregulated at the distal side of leaf explants of *R. aquatica* during plantlet regeneration [2]. *R. aquatica* leaves may have the mechanism to upregulate orthologs of *YUC*, even if the individuals are aged, and this mechanism may be a major contribution to plantlet regeneration. At*ARF7* was temporarily upregulated (Figure 7G), and At*ARF19* was immediately downregulated (Figure 7K). This suggests that the difficulty in regenerating plantlets from leaf explants of *A. thaliana* is attributed to the lack of continuous upregulation of auxin responsive genes. Taken together, the wide intercellular spaces of *A. thaliana* leaves may be related to retaining water and a high photosynthetic efficiency rather than efficient auxin transport.

In this study, we focused not only on molecular mechanisms, but also leaf internal structure as factors affecting plantlet regeneration from leaf explants in nature. Some plant species that can regenerate plantlets (for example, *Sedum*, *Saintpaulia*, and *Peperomia* [1]) develop thick leaves, which may possess packed cells and narrow intercellular spaces, and thus remain viable for longer, similar to *R. aquatica*. Molecular and morphological analyses may effectively explain the viability of leaf explants during plantlet regeneration.

## 4. Conclusions

Leaf explants of *R. aquatica* can survive for long time. This viability may depend on maintaining photosynthetic activity. ABA may also be related to the viability of *R. aquatica* leaf explants by delaying senescence at the transcriptional level. Exogenous JA, a phytohormone that is inferred to upregulate the immune response in *R. aquatica* leaf explants, increased the efficiency of regeneration. Because these results regarding phytohormones only indicate correlations between the viability of leaf explants and the regeneration ability of the plant, further studies will have to reveal their causations. Moreover, our findings, including the difference in internal structure between *R. aquatica* and *A. thaliana,* can be used as a starting point for future studies to understand the fundamental differences between plant species that can or cannot regenerate in nature.

## 5. Materials and Methods

### 5.1. Plant Materials and Plantlet Regeneration

*Rorippa aquatica* (Accession “N” [23]) plants were grown and propagated as previously described [2]. In brief, *R. aquatica* plants were grown for over 50 days in a growth chamber at 30 °C under continuous light and 50 µmol m^−2^ s^−1^ light intensity by fluorescent lamp. Plantlet regeneration was allowed for approximately 2 weeks at 23 °C under continuous light. In addition, *Arabidopsis thaliana* “Col-0” plants were grown at 23 °C for 40 days under continuous light. Leaf explants of *R. aquatica* and *A. thaliana* were placed on wet paper towels in a plastic tray and covered with plastic wrap.

### 5.2. Photosynthetic Activity Measurement

Photosynthetic activity was evaluated by the maximum quantum yield of photosystem II (*F*v/*F*m) and electron transport rate (ETR) using a Mini-PAM (pulse amplitude modulation) portable chlorophyll fluorometer (Walz, Effeltrich, Germany). Minimum fluorescence (*F*o) was obtained from the open photosystem II reaction centers in the dark-adapted state by a low-intensity light (650 nm, 0.05–0.1 µmol photons m^−2^ s^−1^). A saturating pulse of white light (800 ms, 3000 µmol photons m^−2^ s^−1^) was applied to determine the maximum fluorescence with closed photosystem II centers in the dark-adapted state (*F*m) and during illumination with actinic light (*F*m’). The steady-state fluorescence level (*F*s) was recorded during actinic light illumination. The *F*v/*F*m and photosystem II quantum yield (Φ_PSII_) was calculated as (*F*m − *F*o)/*F*m and (*F*m’ − *F*s)/*F*m’, respectively [24]. The relative ETR was calculated as Φ_PSII_ × light intensity (µmol photons m^−2^ s^−1^). Five leaf explants of *R. aquatica* were placed in dark conditions to stabilize the photoresponse for 30 min before measurement of photosynthetic activity in each time point. For regenerating *R. aquatica* plantlets in the dark, leaves of *R. aquatica* were cut into 30 explants and placed on wet paper towels in a plastic tray. The plastic tray was then covered with plastic wrap and aluminum foil, and maintained at 30 °C for 22 days under continuous light conditions. The plastic tray for the light condition was covered with only plastic wrap. The regenerated roots and shoots were counted separately and presented as box plots.

### 5.3. Quantification of Phytohormones

To assess if the phytohormone levels trigger senescence in leaf explants, endogenous ABA and JA levels in *R. aquatica* and *A. thaliana* leaf explants were quantified over time. Furthermore, as *R. aquatica* plantlets are regenerated only at the proximal side of the leaf explant, we hypothesized that the phytohormone levels are different at the distal and proximal sides, and that senescence is more conveniently triggered at the distal side than at the proximal side. Therefore, the phytohormone levels at the distal and proximal sides were quantified separately. The distal and proximal sides from five explants of *R. aquatica* and *A. thaliana* leaf explants were collected separately at different time points, following a previous report [2]. ABA, JA, and SA were extracted following previously described methods [25,26].

For the quantification of IAA and its metabolites, the distal and proximal sides of *R. aquatica* and *A. thaliana* leaf explants were collected separately at different time points as previously described [2]. These were then extracted and detected following a previous report [27].

### 5.4. Photosynthetic Inhibitor and Phytohormone Supplementation

Cultured leaf explants of *R. aquatica* and *A. thaliana* were disinfected with 70% ethanol (*v*/*v*) as previously described [2]. Disinfected leaves were cut into 32 explants and cultured on agar medium (8.0 g L^−1^ agar (Wako, Osaka, Japan)) containing either 25 µM DCMU (alias Diuron, Sigma-Aldrich, St. Louis, MO, USA), 50 µM ABA (Tokyo Chemical Industry, Tokyo, Japan), or 5 µM MeJA (Tokyo Chemical Industry, Tokyo, Japan), at 23 °C under continuous light for 22 days. The control (mock) medium contained dimethyl sulfoxide (DMSO) (Wako, Osaka, Japan). Regenerated roots and shoots were counted separately and presented as box plots.

### 5.5. Analysis of Gene Expression Profiles Using Transcriptome Data

RNA-seq of *R. aquatica* has previously been performed using the NextSeq500 sequencing platform (Illumina, CA, USA) [2]. Transcriptome data of *R. aquatica* have previously been reported [2]. RNA-seq of *A. thaliana* leaf explants was performed at 0 h, 1 h, 3 h, 6 h, 9 h, 12 h, 15 h, 18 h, 21 h, 1 d, 2 d, 3 d, and 6 d after leaf cutting for the first time in this study, following the same procedure as for *R. aquatica* explants at 0 h, 1 h, 3 h, 6 h, 9 h, 12 h, 15 h, 18 h, 21 h, 1 d, 2 d, 3 d, 6 d, 8 d, 10 d, and 12 d after leaf cutting [2]. RNA was extracted from approximately 3–5 mm square pieces of leaf explant of *A. thaliana*. Extracted RNA samples (*n* = 3) were assessed for quality (integrity number ≥ 0.8) using the Agilent RNA6000 Nano assay (Agilent, CA, USA). Libraries were prepared using the Illumina TruSeq^®^ Stranded RNA LT Kit (Illumina, CA, USA) and quantified using the QuantiFluor^®^ dsDNA System (Promega, WI, USA). The quality of libraries was checked using the Agilent High Sensitivity DNA Assay (Agilent, CA, USA). Libraries were pooled and sequenced on the NextSeq500 sequencing platform (Illumina, CA, USA). The obtained 75-bp single-end reads were mapped to the genome sequence of *A. thaliana*. Gene expression profiles with *A. thaliana* genome sequence (TAIR10) were plotted using ‘ggplot2′ and ‘gridExtra’ packages in R.

### 5.6. Observation of the Internal Structure of Rosette Leaves

To observe the internal structures of leaves, *R. aquatica* and *A. thaliana* were grown for 50 days and 40 days, respectively. To prepare transverse sections, *R. aquatica* and *A. thaliana* rosette leaves were obtained after embedding in Technovit 7100 resin (Kulzer, Hanau, Germany), as previously described [3,28]. Sectioned samples were stained with 0.1% toluidine blue (*w*/*v*) (*n* = 3).

### 5.7. Statistical Analysis

The experimental design was completely randomized. For measurement of photosynthetic activity and quantification of endogenous phytohormones, data from at least four independent experiments were averaged. To quantify the regenerated roots and shoots, data from at least 30 leaf explants were analyzed using Student’s *t*-test, where *p* < 0.05 indicates significance. *p* values in graphs indicated exact values.

### 5.8. Sequence Data

The RNA-seq data of *R. aquatica* are available from the DNA Data Bank of Japan Sequenced Read Archive (DRA006777). The RNA-seq data of *A. thaliana* are available from the DNA Data Bank of Japan Sequenced Read Archive (DRA010684).
